# Response to Growth Hormone Treatment in a Patient with Insulin-Like Growth Factor 1 Receptor Deletion

**DOI:** 10.4274/jcrpe.4456

**Published:** 2017-12-15

**Authors:** Ranim Mahmoud, Ajanta Naidu, Hiba Risheg, Virginia Kimonis

**Affiliations:** 1 University of California, Department of Pediatrics, Division of Genetics and Genomic Medicine, Irvine, California, USA; 2 University of California, Department of Pediatrics, Division of Endocrinology, Irvine, California, USA; 3 Children’s Hospital of Orange County, Orange, California, USA; 4 Laboratory Corporation of America/Dynacare, Department of Cytogenetics, Seattle, Washington, USA

**Keywords:** Growth hormone therapy, growth hormone receptor, short stature, 15q deletion, duplication 4q

## Abstract

We report a six-year-old boy who presented with short stature, microcephaly, dysmorphic features, and developmental delay and who was identified with a terminal deletion of 15q26.2q26.3 containing the insulin-like growth factor receptor (IGF1R) gene in addition to a terminal duplication of the 4q35.1q35.2 region. We compare our case with other reports of deletions and mutations affecting the IGF1R gene associated with pre-and postnatal growth restriction. We report the dramatic response to growth hormone therapy in this patient which highlights the importance of identifying patients with IGF1R deletion and treating them early.

**What is already known on this topic?**

Insulin-like growth factor receptor (IGF1R) mutations are suspected in patients born small for gestational age with normal endocrine and metabolic workup. Single nucleotide polymorphism microarray may help to identify those patients with deletions that include the IGF1R gene. Early initiation of growth hormone (GH) treatment with higher dosing than used in growth hormone deficiency may lead to significant improvement in growth parameters. Reports in the literature reveal variable rates of response to GH treatment.

**What this study adds?**

We report the dramatic response to growth hormone therapy in this patient which highlights the importance of identifying patients with IGF1R deletion and treating them early.

## INTRODUCTION

Fetal growth is dependent on maternal, placental, fetal, and environmental factors. The mechanisms of human fetal growth remain unknown in many cases. Insulin-like growth factor 1 (IGF-1) has a crucial role in the regulation of pre- and postnatal growth. It promotes growth during embryogenesis and postnatal life via DNA synthesis stimulation, cell proliferation, cellular differentiation, and also by increasing glucose uptake in adipose tissue and muscle cells (1). The role of IGF-1 is not limited to growth promotion and weight gain but is also important in promoting brain and inner ear development (2,3).

IGFs are produced primarily in the liver in response to growth hormone (GH), while their metabolic effects are mainly due to their binding of GH with its receptors on target cells.

The gene coding the IGF1 receptor (IGF1R), located on the long arm of chromosome 15, is involved in somatic development and glucose metabolism. Terminal microdeletion of the long arm of chromosome 15 is a rare cause of short stature. Most cases have been associated with pre- and postnatal growth restriction, microcephaly, and developmental delay (4,5).

Herein, we report a six-year-old male who presented with developmental delay and short stature with a terminal deletion of 15q26.2q26.3. This region includes the IGF1R gene and a terminal duplication of the 4q35.1q35.2 identified by single nucleotide polymorphism (SNP) microarray analysis. This report emphasizes the important role of SNP microarrays in investigating short stature where significant cognitive impairment or marked dysmorphism are not prominent features. It also elucidates the benefit of GH therapy in patients with IGF1R deletions and highlights the importance of early diagnosis and treatment of these patients.

## CASE REPORTS

**Figure 1:**
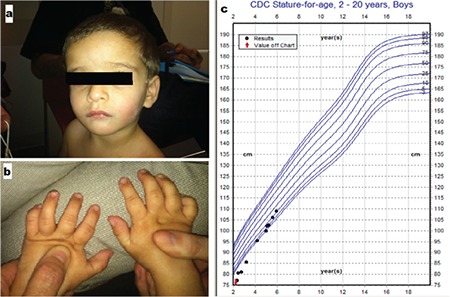
Photograph showing frontal bossing, low-set ears (a), and marked clinodactyly of both fifth digits (b). Height for age curve of the patient showing that the patient’s height was below 3rd percentile for age until growth hormone (GH) treatment was started at the age of 41 months. Following institution of GH therapy, catch up growth was noted (c) CDC: Center for Disease Control and Prevention

We report a male born at full term by cesarean section following a pregnancy complicated by oligohydramnios and severe intrauterine growth retardation (IUGR). At birth, his length was 43.18 cm (Z score -3.2) and weight was 2102 grams (Z score-3). His history was significant for gastroesophageal reflux during the first three months of life treated by ranitidine. On review of his milestones, he was smiling by six months, sitting at six months, standing at 20 months, and walking at 21 months. He had his first teeth at one year of age. He said his first words at 20 months. He initially presented to the genetics clinic at the age of 30 months for short stature. On physical examination, his height was 60.2 cm (Z score -9.3), weight was 8.49 kg (Z score -4.7), and head circumference was 45 cm (Z score -3). Dysmorphic features including mild frontal bossing, low-set ears, and marked clinodactyly of his fifth digits (Figure 1) were suggestive of Russell-Silver syndrome. However, his proportionate head size and developmental delay were inconsistent with this diagnosis. His chest, abdomen, genitourinary system examination, and neurologic findings were unremarkable. Initial laboratory evaluation showed normal thyroid function studies, IGF binding protein 3 (IGFBP3) was 2.4 mcg/mL (normal range 1.1-5.2), and IGF1 25 ng/mL (normal range 30-174). His biochemical workup results were within normal ranges. Bone age radiograph revealed the presence of normally shaped phalangeal epiphyses with a significantly delayed bone age 2 standard deviation below his chronological age. Abdominal ultrasound, echocardiography, and cerebral magnetic resonance imaging findings were normal. Developmental evaluation was done at the age of three years using the Wechsler Preschool and Primary Scales of Intelligence 4th edition and Vineland Adaptive Behavior Scales 2nd edition. His general cognitive ability was in the low range of intellectual functioning as measured by the Full Scale Intelligence Quotient=72, 3rd percentile. His Verbal Comprehension Index was 84, in the below average range. His speech was normal for rate, rhythm, and prosody but with multiple articulation errors. Vineland Adaptive Behavior Composite Score was 61 (<1 percentile). He was observed to be easily distracted and was slightly hyperactive.

**Figure 2:**
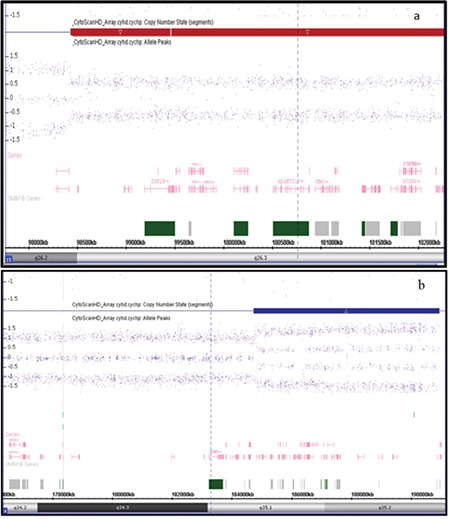
PSingle nucleotide polymorphism microarray showing the 4.09 Mb terminal deletion of 15q26.2->15qter arr [hg19] 15q26.2q26.3 (98,434,315-99,459,796) x1 (a) and the 6.41 Mb terminal duplication of 4q35.1->4qter arr[hg19] 4q35.1q35.2 (184,738,819-190,957,473) x3 (b)

SNP microarray analysis was performed using the Affymetrix Cytoscan HD platform. 250 ng of total genomic DNA extract was digested with NspI and then ligated to NspI adaptors, respectively, and amplified using Titanium Taq with a GeneAmp PCR System 9700 (Applied Biosystems, Foster City, California). Polymerase chain reaction products were purified using AM Pure beads (Agencourt Biosciences, Beverly, Massachusetts) and quantified using NanoDrop 8000 (Thermo Fisher, Wilmington, Delaware). Purified DNA was fragmented and biotin labeled and hybridized to the Affymetrix Cytoscan HD Gene Chip. The data were analyzed using Chromosome Analysis Suite. The analysis was based on the GRCh37/hg19 assembly. SNP microarray identified a 4.09 Mb terminal deletion of 15q26.2q26.3 [arr[hg19] 15q26.2q26.3(98,434,315-99,459,796)x1] and included the IGF1R gene. Microarray also showed a 6.41 Mb terminal duplication of the 4q35.1q35.2 [arr[hg19] 4q35.1q35.2(184,738,819-190,957,473)x3] (Figure 2). The duplicated region comprised numerous genes of uncertain clinical significance, this region being flanked by ENPP6 and DBET genes.

Daily subcutaneous GH therapy was started at the age of 41 months with a mean dose of 35 ug/kg/day. His follow-up growth parameters 6 months later revealed improvement; his weight was 11.4 kg (Z score -3) and his height was 87.4 cm (Z score -3.7). The dose was increased to 40 ug/kg/day. His growth velocity just prior to GH treatment was 1.84 cm/year. His follow-up after one year shows improvement in his growth parameters. His weight was 14.9 kg (Z score -2), height was 99.8 cm (Z score -1.89), and head circumference was 46.5 cm (Z score -2). Over the past 15 months, he has received consistent treatment which is reflected in his post-treatment growth velocity of 8.47 cm/year. His height and weight is now at the 10th percentile for age. Additionally, there is an improved IGF1 level of 312 ng/mL and improved IGFBP3 level of 3.4 mg/L (normal range 1.5-3.4 mg/L).

The family history was significant for learning problems in the mother. Maternal height was 175 cm and paternal height was 180 cm, yielding a mid-parental target height of 184 cm. There was no consanguinity reported and no other significant medical history. Fluorescence in situ hybridization testing of the father was negative and permission for maternal testing could not be obtained. Informed consent was obtained from the family.

## DISCUSSION

In this report, we describe a patient with IUGR, short stature, microcephaly, developmental delay, and mild facial dysmorphism. SNP microarray demonstrated a terminal deletion of 15q26.2q26.3 containing the IGF1R gene and terminal duplication of the 4q35.1q35.2.

Although similar cases have been previously reported, clinical features in patients with IGF1R abnormalities have not been well-defined. Based on our report and previous studies, they all share the common features of growth retardation and microcephaly. However, mental performance is not essentially affected (6,7). Cardiac, lung, gastrointestinal, and renal anomalies have been reported in some patients (8,9). Choi et al (10) have reported a case with IGF1R haploinsufficiency due to deletion of the chromosome 15q26.2 in association with short stature, coarctation of the aorta, right cryptorchidism, left multicystic dysplastic kidney disease, and dysmorphic features including microcephaly, bilateral ptosis, strabismus, long palpebral fissure, and clinodactyly. These additional clinical features were mostly linked to other genes in the same region affected by the deletion (11,12).

As previously reported, multiple family members may show similar features of growth retardation and microcephaly, and molecular analysis of the affected family members has revealed similar IGF1R mutations (13,14,15). Several mutations have been so far described affecting 15q locus leading to IGF1R dysfunction. The first case of an interstitial deletion of chromosome 15 was reported by Fryns et al (16). Terminal deletions of 15q26 have been described by others (5,10). Clinical features and underlying mutations of reported cases are summarized in Table 1 (5,6,10,12,13,15,17,18,19).

In addition to 15q26.2q26.3 deletion, our patient was shown to have a terminal duplication of the 4q35.1q35.2. It is known that individuals with 4q duplication may exhibit variable phenotypes such as microcephaly, brachycephaly, slanting eyes, low-set ears, retromicrognathia, small or missing thumb, and congenital cardiac defects, the variability attributable to the specific band duplication (20). Our patient appears to have a milder phenotype than the reported case with a duplication of 4q24qter (21) and the siblings with duplication of 4q31-35 (22). It is possible that his phenotype is modified by the deletion of chromosomal 15q26.2q26.3 region or other unknown genetic/epigenetic factors.

Growth retardation caused by IGF1R haploinsufficiency was successfully treated with GH in some of the earlier reports (5,17). However, full catch-up was not achieved. Although the response to GH in patients with IGF1R mutations was inconsistent in some of the available reports (18,19), our patient has shown a promising initial response with an annual height velocity of 12.4 cm/year after 1.5 years of GH treatment. Similarly, Walenkamp et al (5) reported a 15-year-old girl with IGF1R deletion who responded dramatically to GH therapy started at the age of five years. A normal adult height was eventually achieved in this patient (5). In contrast, no height gain was observed in a 13.5-year-old girl reported by Inagaki et al (15) who received recombinant human GH for 6 months. Abuzzahab et al (6) reported a 4.5-year-old female whose growth rate increased to 7.2 cm per year during the first year of GH therapy and which slowed down after GH therapy discontinuation. Her height velocity increased to 6.5 cm per year when treatment was resumed (6).

The positive effect of GH treatment may be explained by its direct stimulatory effect or marked elevation of IGF1 levels that could stimulate partially insensitive receptors. It is recommended to start treatment with a small dose and titrate the dose up based on treatment response. Choi et al (10) proposed that higher doses of GH 0.217 mg to 0.7 mg/kg were needed to achieve optimal response. Variable response to GH may be explained by variable responsiveness of abnormal IGF1R resulting from the different underlying mutations. Variable mutation extent may also explain variability of the mutant receptor response to GH therapy.

Given the detrimental consequences of IGF1 unresponsiveness on both somatic growth and psychomotor development, the diagnosis of IGF1R mutation/deletion should be considered in a child with growth retardation and otherwise negative laboratory studies. Although rare, delay in treatment could seriously impact the growth and development pattern (15).

In conclusion, IGF1R mutations must be suspected in patients born small for gestational age with normal endocrine and metabolic workup results. SNP microarray may help to identify those patients with deletions that include the IGF1R gene. Early initiation of GH treatment with higher dosing than used in GH deficiency may lead to significant improvements in growth parameters and catch up growth.

## Figures and Tables

**Table 1:**
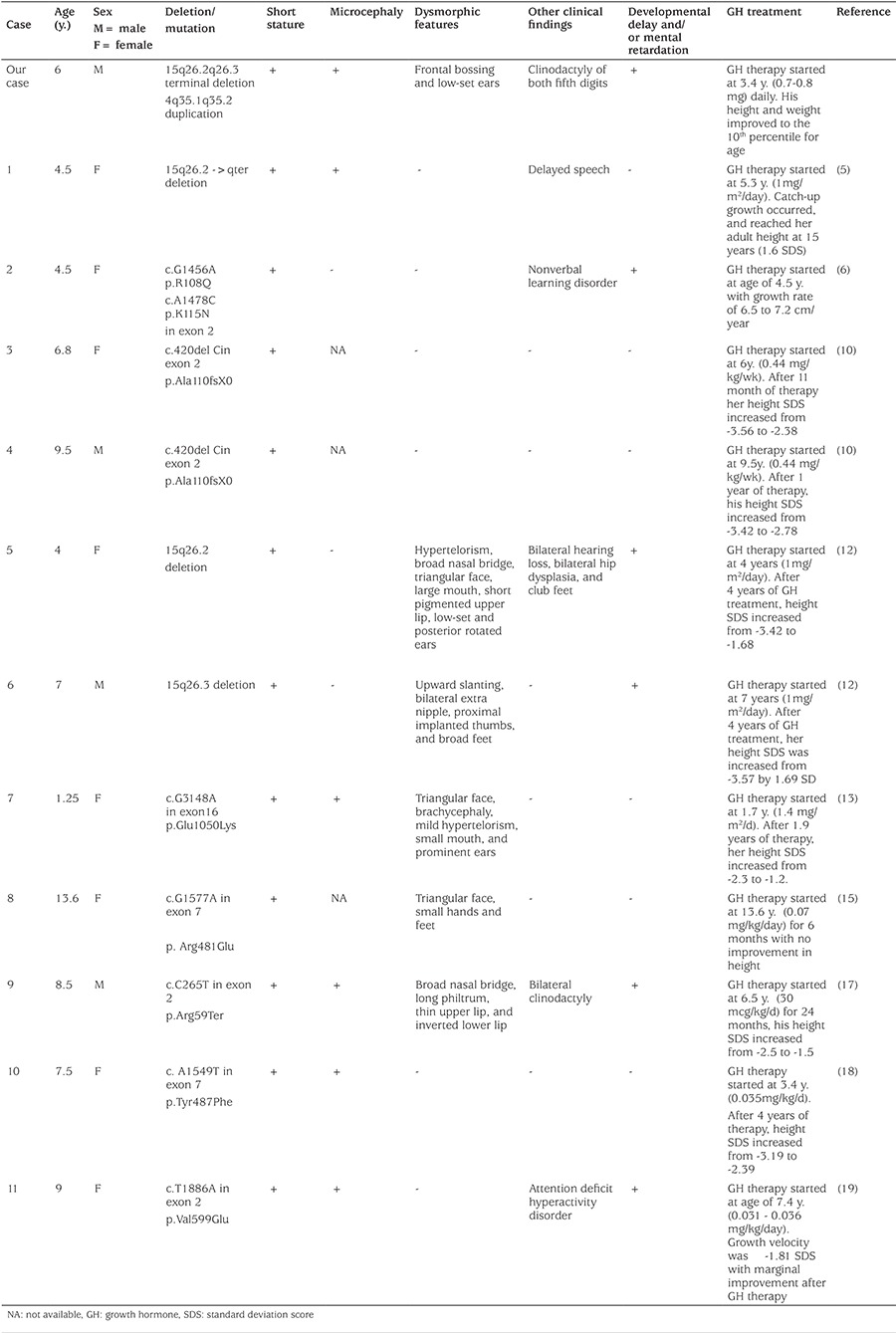
Clinical and other features of reported cases
